# A dataset for tactile textures on uneven surfaces collected using a BioIn-Tacto sensing module

**DOI:** 10.1016/j.dib.2025.111312

**Published:** 2025-01-20

**Authors:** Maliheh Marzani, Soheil Khatibi, Ruslan Masinjila, Vinicius Prado da Fonseca, Thiago Eustaquio Alves de Oliveira

**Affiliations:** a Department of Computer Science, Lakehead University, Orillia, ON, Canada; b Department of Computer Science, Memorial University of Newfoundland, St. John's, NL, Canada

**Keywords:** Texture classification, Dynamic exploration, Tactile sensor, Machine learning

## Abstract

Effective human-like manipulation in robots depends on their capacity to recognize and identify textures in different environments. In unpredictable environments, robots with tactile sensors will have to identify textures through touch-related features. To advance research in texture classification, a comprehensive dataset capturing the physical interactions between a tactile-enabled robotic probe and various textures is necessary. As a result, we are driven to create a dataset from the signals collected by a bioinspired multimodal tactile sensing module, as a robotic probe dynamically makes contact with 12 different tactile textures. This dataset includes signals for pressure, acceleration, angular rate, and magnetic field variations, all captured by sensors embedded within the flexible structure of the sensing module. The pressure signals and the signals from the other sensors were sampled at a rate of 130 Hz. Each texture was explored 25 times, with each exploration involving a sliding motion along the uneven surface, tangential to the surface where the texture was bonded. The dataset comprises a total of 300 exploratory episodes. The tactile texture dataset applies to various projects in object recognition and robotic manipulation, making it particularly valuable for tasks involving tactile texture reconstruction and recognition. Additionally, this dataset offers opportunities to study time series properties generated by the robotic sliding motions during tactile texture exploration.

Specifications Table

**Table:** 

Subject	Robotics
Specific subject area	Tactile texture sensing, perception and recognition.
Type of data	CSV files, python scripts for parsing and pre-processing.
Data collection	This dataset provides tactile sensor data captured using a BioIn-Tacto multi-modal tactile sensing [1,2] module mounted on the end-effector of an OpenManipulatorX [3]. The module provides data from a nine degrees-of-freedom Magnetic, Angular Rate, and Gravity (MARG) system, along with deep pressure data from a barometer encased in polyurethane rubber. The barometer is mounted in the base of the module to capture deformation. The sensing module is mounted on a holder designed to simulate human-like texture exploration. Each texture is glued onto a curved surface and placed on a table. The OpenManipulatorX robotic arm, equipped with the sensing module, slides the sensor along the surface by performing a predefined trajectory that follows the curvature of the surface. During each exploratory episode, the arm moves the sensing module along this trajectory, ensuring consistent contact with the texture to collect tactile data. Further details regarding the design of the BioIn-Tacto sensing module used in the data acquisition can be found in [2]. Fabrication details for the BioIn-Tacto can be found in [1].
Data source location	Institution: Lakehead University, City/Town/Region: Orillia, ON Country: Canada
Data accessibility	Repository name: Dynamic Tactile Data of Textures On Uneven Surfaces Data identification number: 10.17632/khpwng8thh.1 Direct URL to data: https://data.mendeley.com/datasets/khpwng8thh/1 Instructions for accessing these data: The full database can be downloaded from the Mendeley Data Repository mentioned above end in [4].
Related research article	none

## 1 Value of The Data

• This dataset is valuable for advancing the development of robotic tactile sensors, enabling robots to interact with objects through touch. These interactions can be dynamic, generating signals that vary over time as the sensor navigates across uneven surfaces. Unlike previous datasets, which often focus on flat surfaces [[5], [6], [7], [8]] our dataset emphasizes dynamic tactile data collected from a variety of commonly encountered textures across uneven surfaces, enhancing its applicability to real-world tasks [9]. This approach builds upon prior work in dynamic tactile sensing [10], employing a robotic arm instead of an XY plane for exploration, expanding beyond synthetic textures [11], macroscopic profiles [12], and benefiting from advancements in tactile sensing technologies for robots [13], contributing to a richer understanding of real-world uneven textures.
• This dataset also addresses the gap in the availability of tactile perception data, as previous research [14] has not consistently made their datasets publicly available. By ensuring access to this dataset, we aim to support ongoing research and development in this area. The dataset proposed here complements [10] by providing data from uneven surfaces, and is publicly available at https://data.mendeley.com/datasets/khpwng8thh/1.
• Researchers in the field of robotics can benefit from this dataset, using it to explore and enhance texture recognition and manipulation capabilities on uneven surfaces. Beyond robotics, the dataset may also attract researchers from other fields. For example, the same type of data has been used in haptic surface reconstruction [15], and tactile object recognition [16], showcasing its adaptability for a range of applications.
• In [17], the authors explore tactile texture recognition using neural networks, but their dataset has yet to be made available. Similarly, [18] presents a texture dataset using accelerometer data, but only examines textures in one direction. Our dataset complements this by exploring 2D textures on uneven surfaces using multiple sensing modalities.
• These data can be used to develop and refine machine learning algorithms, improving accuracy and efficiency in texture classification for robotic tactile sensing systems. Our dataset provides insight into how texture recognition can be enhanced by using data from uneven surfaces, and has already been used in [8] to classify textures in different exploratory scenarios.
• Researchers can also leverage this dataset as a benchmark to evaluate the performance of machine learning models on sequential tactile data, driving advancements in texture classification and tactile perception on complex, uneven surfaces.
• The dataset presented in this study is designed to support diverse applications in tactile sensing and texture recognition. Researchers can apply the methods proposed in [[7], [8], [9],^19^,^13^,^14^], and [17] to analyze this dataset, leveraging advanced techniques for texture classification and tactile perception in dynamic environments. This dataset, which focuses on textures on uneven surfaces, presents a more challenging scenario than datasets focusing on flat surfaces, offering opportunities to extend and test the adaptability of existing methods. Furthermore, it complements the dataset published in [10], providing a richer resource for researchers aiming to improve tactile texture recognition and related machine learning tasks.

## 2 Background

The objective of generating this dataset is to provide a comprehensive collection of data that explores the recognition of tactile textures in dynamic exploration scenarios over uneven surfaces. The dataset was acquired using the OpenManipulatorX robotic arm equipped with a BioIn-Tacto multi-modal tactile sensing module. By incorporating data from pressure, Magnetic field, Angular Rate, and Gravity (MARG) sensors, the dataset aims to support research on machine learning methods for texture classification, particularly in more complex and realistic environments with uneven surfaces.

This dataset can also be used to address research questions related to few-shot, zero-shot, and sequential learning for tactile texture classification. Additionally, the dataset increases the availability of MARG data for texture recognition tasks, aiding in the development of machine learning models for tactile sensing.

Tactile texture classification from sliding motions over uneven surfaces is a crucial precursor when a robot interacts with surfaces, whether to manipulate, modify, or place objects on them. Texture information may also complement object identification tasks, as objects with similar shapes, stiffness, and weights may differ in their surface textures and microscopic structures, contributing to more accurate recognition and manipulation.

## 3 Data Description

The dataset contains raw sensor measurements stored in CSV files. It also includes sample scripts that read the CSV files and use Python's Pandas library to access their data. The data files are organized in a specific folder structure and contain multiple readings for each texture and exploratory episode. The dataset contains raw data recorded from tactile measurements for different textures, stored in CSV format. Table 1 shows the dataset folder and subfolder descriptions.

**Table 1:** Sample contractions and their possible expanded forms from the contractions dictionary.

*Folder Name*	*Description*
Data/	Main folder containing all data subfolders.
Data/0_Raw/	Folder with raw barometric and IMU data for 12 textures (T1 to T12), each with 25 episodes.
Data/1_Merged/	Contains merged barometric and IMU data for each texture and episode.
Data/2_Trimmed/	Preprocessed, trimmed data for each texture and episode.
Data/3_Normalized/	Normalized data for consistent scaling across episodes.
Data/4_Windowed/	Windowed data segments for analysis.
Scripts/	Contains all Python scripts for data preprocessing.

The folder structure for the **Data** folder, specifically the raw data in the 0_Raw/ subfolder, follows this hierarchical structure:

• **Folder**: T1 to T12 (each representing a texture)
  ○ **Subfolder**: 1 to 25 (each representing an exploratory episode)
    ■ **CSV file**: baro.csv (contains barometric data)
    ■ **CSV file**: imus.csv (contains IMU data)

Once the two CSV files (baro.csv and imus.csv) are merged, they form a single merged_data.csv file for each episode, stored in the 1_Merged/ subfolder.

In the 4_Windowed/ subfolder, the folder structure evolves to store windowed data:

• **Folder**: T1 to T12 (each representing a texture)
  ○ **Subfolder**: 1 to 25 (each representing an exploratory episode)
    ■ **Folder**: windows/ (contains windowed data)
      ■ **Subfolder**: 128/, 256/, 512/ (represent different window sizes)
        ■ **Files**: Various files representing each window, containing segments of the merged data for analysis.

Additionally, the windowed data for each episode is converted into .npy format for efficient processing, with the .npy files stored directly in the **Data/** folder:

• **X_windows_128.npy, X_windows_256.npy, X_windows_512.npy**: Numpy arrays containing windowed data for different window sizes.
• **y_labels_128.npy, y_labels_256.npy, y_labels_512.npy**: Numpy arrays containing labels corresponding to the window sizes.

Scripts/

The **Scripts/** folder contains the Python and Shell scripts used to automate the data preprocessing pipeline:

• **merge.py**: Merges the barometric and IMU data into a single CSV file for each exploratory episode.
• **trim.py**: Trims the merged data based on predefined trimming points, removing unnecessary data.
• **normalize.py**: Normalizes the trimmed data to ensure consistency in scale across all episodes.
• **window_creator.py**: Segments the normalized data into smaller windows (128, 256, 512) for analysis.
• **npy_creator.py**: Converts the windowed CSV files into .npy format for efficient loading and machine learning tasks.
• **run.sh:** The **run.sh** shell script sequentially automates the entire preprocessing pipeline by running all the key scripts in order:
  1. merge.py
  2. trim.py
  3. normalize.py
  4. window_creator.py
  5. npy_creator.py

This allows for end-to-end processing of barometric and IMU data, from raw sensor measurements to final windowed .npy files ready for machine learning applications. Table 2, Table 3 present five consecutive raw readings from CSV files of the barometer and MARG sensors, respectively.

**Table 2:** An example of five consecutive sensor readings from the baro.csv file in 0_Raw/T1/1. The *field.baros0.baro_level* column contains pressure measurements from the analog-to-digital converter readings for the barometer sensors.

*%time*	*field.baros0.baro_level*
1716394286443049724	362.0
1716394286450561998	364.0
1716394286458469507	364.0
1716394286466198732	364.0
1716394286473878483	363.0

**Table 3:** An example of five consecutive sensor readings from the CSV file 0_Raw/T1/1/imus.csv. The imu_ax, imu_ay, and imu_az columns display the accelerometer readings in mm/s². The imu_gx, imu_gy, and imu_gz columns show the angular velocity measurements in rad/s. The imu_mx, imu_my, and imu_mz columns display the measurements of the magnetic field amplitude surrounding the sensor in the x, y, and z directions of the sensor reference frame. These measurements are in gauss.

%time	field.imus0.ax	field.imus0.ay	field.imus0.az	field.imus0.gx	field.imus0.gy	field.imus0.gz	field.imus0.mx	field.imus0.my	field.imus0.mz
1716394286443149969	-2.294244	1.282095	9.214684	0.161100	-0.000400	-0.003927	-0.145020	0.482727	-0.553772
1716394286450662066	-1.527501	0.729633	9.375693	0.211993	0.057149	-0.030026	-0.145020	0.482727	-0.553772
1716394286458575414	-1.366491	0.636259	9.346365	0.118558	0.024916	-0.002100	-0.145020	0.482727	-0.553772
1716394286466330864	-1.894412	1.686117	9.245210	0.110729	-0.008882	0.055057	-0.145020	0.482727	-0.553772
1716394286473972316	-1.485602	0.272340	9.427169	0.186155	0.019044	0.000902	-0.145203	0.603333	-0.525024

## 4 Experimental Design, Materials and Methods

**Fig. 1:**
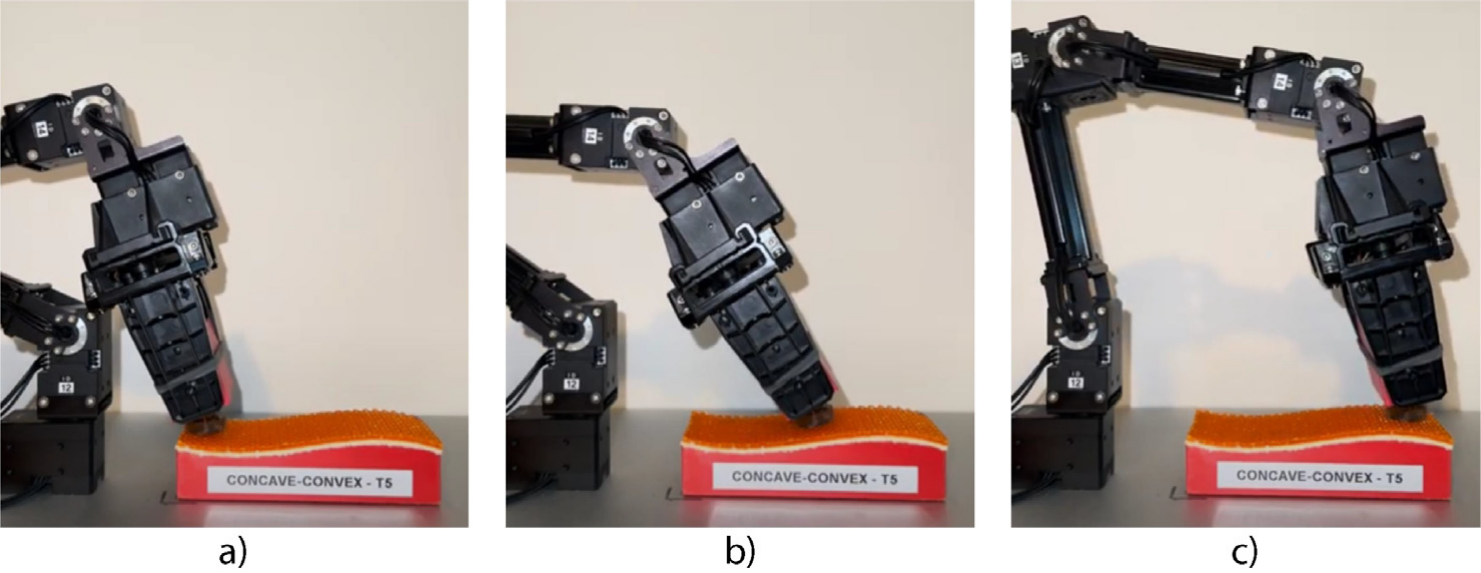
Overview of the OpenManipulatorX robotic arm with the sensing module mounted, collecting data on an uneven surface with a textured material: a) The robot at the beginning of the exploration path; b) The robot at the midpoint of the path; c) The robot at the end of the exploration path

The experimental design employed an OpenManipulatorX robotic arm equipped with a specialized multi-modal sensing module. The sensing module, mounted on the robotic arm, captured tactile data by sliding along various textures placed on curved surfaces. Unlike previous setups using an XY-recorder, the OpenManipulatorX was responsible for executing controlled movements during data collection, allowing the sensor to follow predefined trajectories along the uneven surfaces. An overview of the data collection setup is presented in Fig 1.

**Fig. 2:**
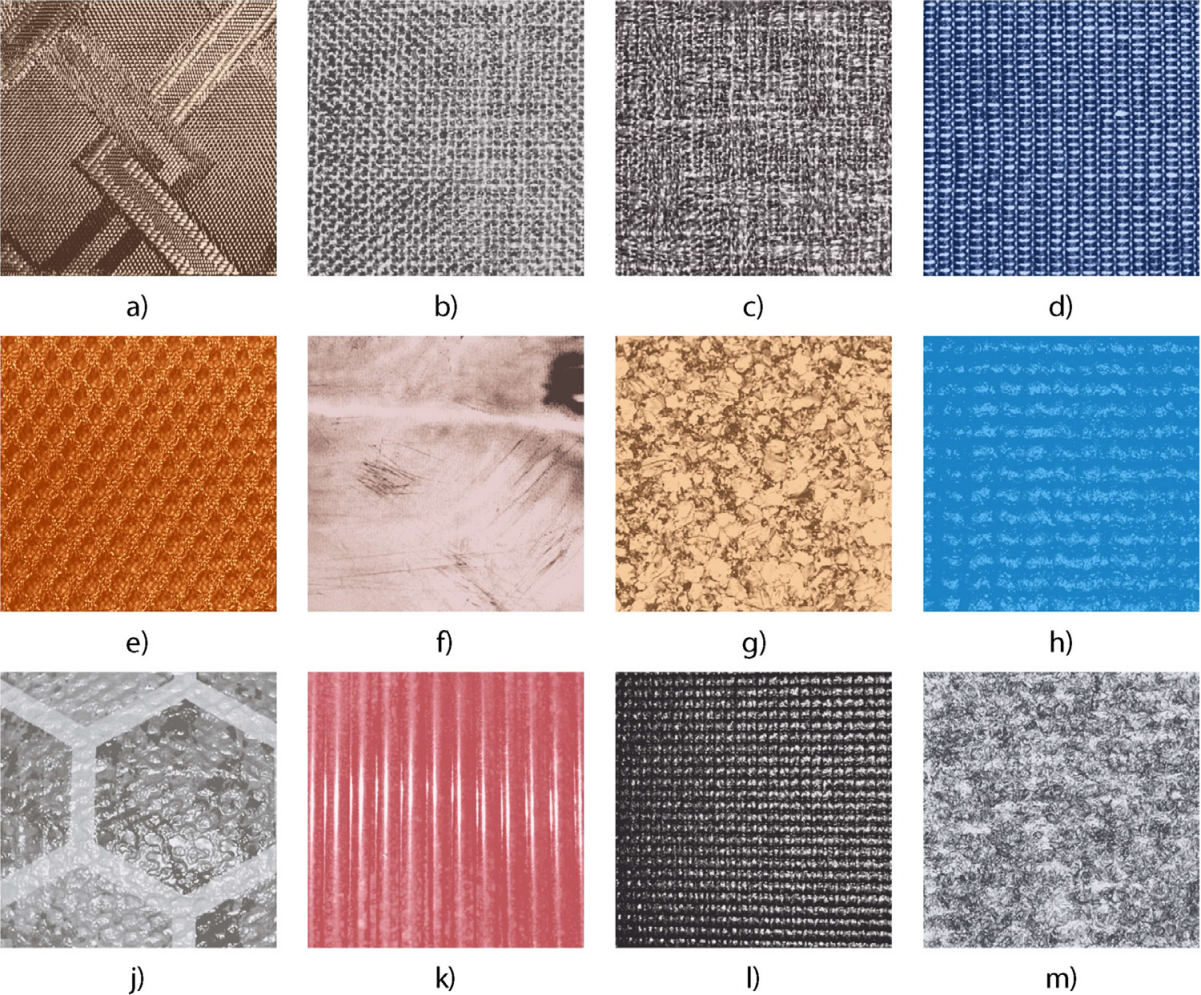
The 12 textures used in the experiments: a) brocade fabric; b) open weave cotton; c) tight weave cotton; d) mesh cotton; e) honeycomb fabric; f) embossed plastic; g) wood; h) silicone mesh; i) reptile-patterned leather; j) ridged polymer; k) mesh leather; l) carpet wool

The materials used in this experimental setup included the OpenManipulatorX robotic arm equipped with a sensing module that recorded data from a range of sensors, including pressure, Magnetic field, Angular Rate, and Gravity (MARG). The textures explored in the experiment consisted of diverse items such as brocade fabric, open weave cotton, tight weave cotton, mesh cotton, honeycomb fabric, embossed plastic, wood, silicone mesh, reptile-patterned leather, ridged polymer, mesh leather, and carpet wool. These textures were securely mounted on curved surfaces to simulate realistic tactile exploration environments. Fig 2 illustrates the textures used for data collection by the robotic arm.

During the experiment, the robotic arm moved along the surface of each texture, following predefined trajectories to ensure consistent and controlled exploration. The arm was programmed to slide the sensing module over the textures, capturing detailed measurements related to pressure, acceleration, angular rate, and magnetic fields. The barometer embedded in the sensing module provided deep-pressure data, capturing surface deformation as the sensor made contact with the textures.

**Fig. 3:**
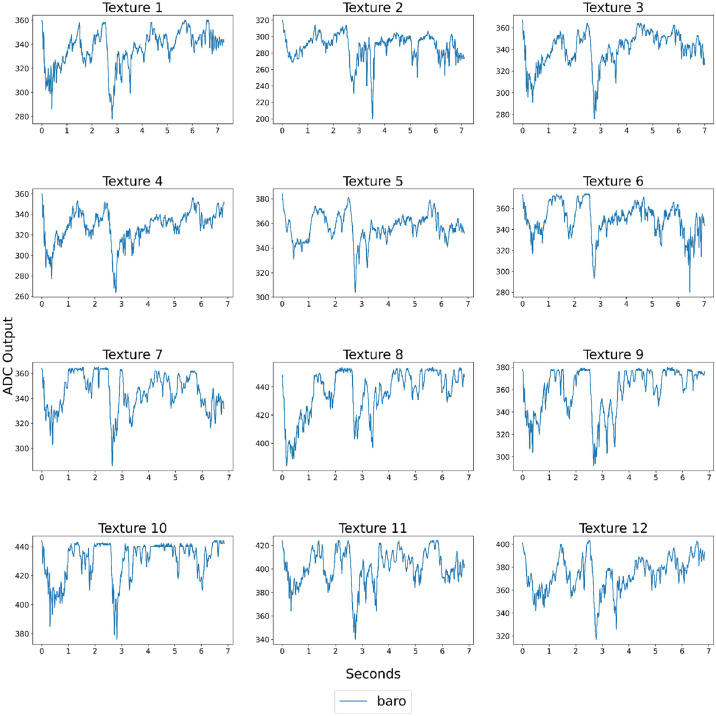
Sample of the barometer data for each texture explored using the OpenManipulatorX.

**Fig. 4:**
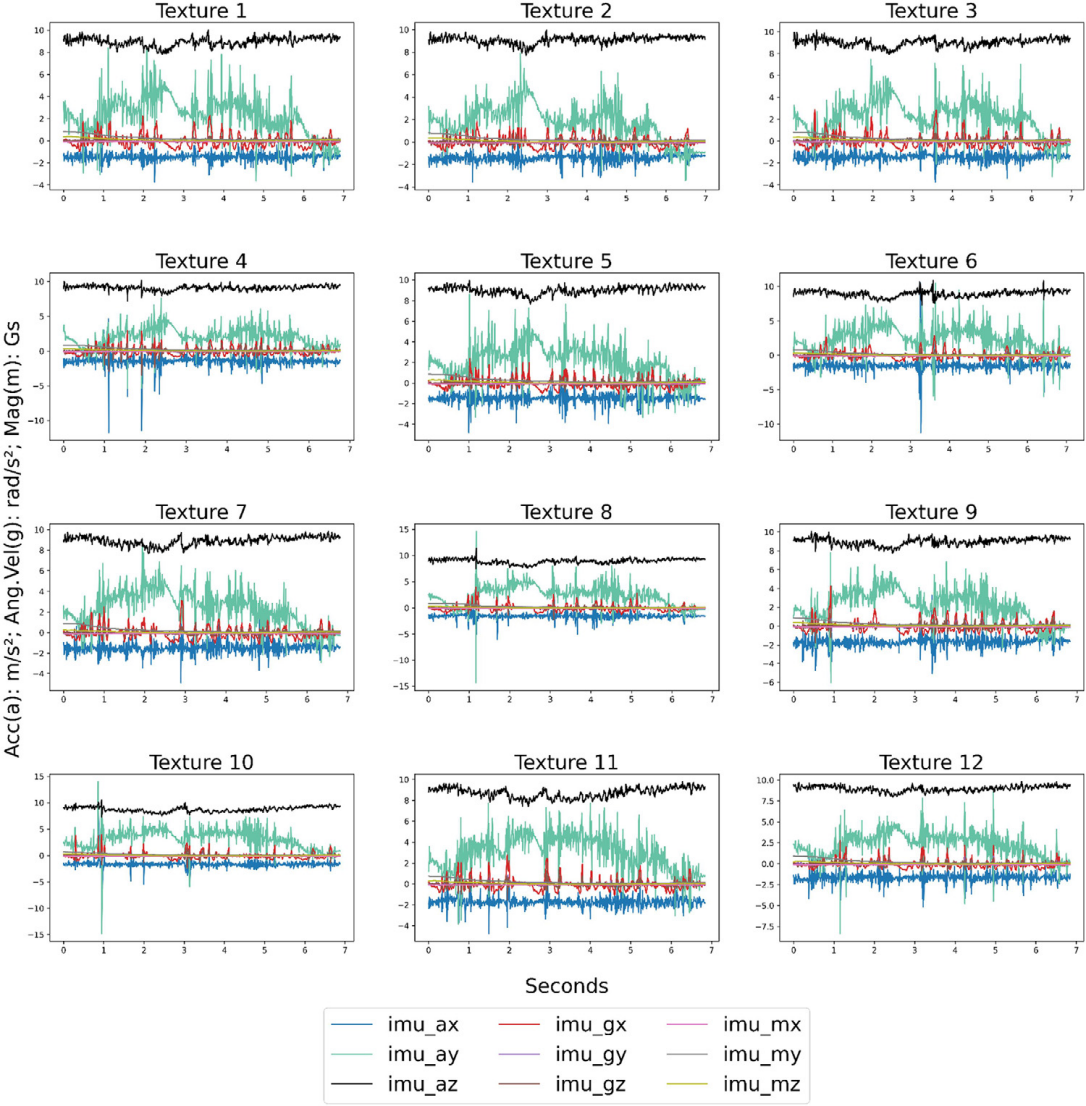
Sample of the MARG data for each texture explored using the OpenManipulatorX.

The data collection process was fully automated using the OpenManipulatorX, with control over the arm's movements and sensing tasks being executed through a combination of hardware components, including the robotic arm, sensing module, and relevant software. This setup ensured a systematic approach to gathering tactile data, which can be utilized for texture recognition, machine learning tasks, and robotic manipulation studies. Fig. 3, Fig. 4 display sample data collected by the pressure sensor and MARG system as the robotic arm glides over the textures.

To ensure the reliability and repeatability of the data acquisition process, the experimental environment was carefully controlled to minimize external variability. The room temperature was maintained at a constant 22°C throughout the data collection process, and the setup was isolated to prevent external vibrations from affecting the robotic manipulator or the textures being explored. Each texture was bonded to a 3D-printed surface, with all surfaces printed using the same 3D printer to maintain consistency. A single coat of contact cement was used to adhere the textures, ensuring even bonding across all samples.

The robotic arm's movements were controlled and validated to guarantee precise and repeatable trajectories during each exploratory episode. Waypoints for the sliding trajectory were automatically calculated using the methodology proposed in [20], which allowed for consistent trajectories across all 25 exploratory episodes per texture. This rigorous approach ensured a high level of repetition consistency and minimized deviations in data acquisition.

During data acquisition, strict quality control measures were implemented to ensure the integrity and reliability of the dataset. We followed the procedures outlined for the BioIn-Tacto sensing module, which are described and made available open-source in [1]. These procedures included zeroing out the accelerometers, gyroscopes, and barometric sensors before each data collection session to prevent signal drift and maintain accurate measurements.

To ensure time-aligned readings across all sensor modalities, including pressure and MARG sensors, we synchronized the data streams using the Robot Operating System (ROS) framework [21]. Additionally, measures were taken to secure the robotic arm and the sensing module to a stable, sturdy table, and the module was carefully fastened to eliminate any movement that could compromise data consistency.

After the data was collected, we conducted manual checks to identify and address any missing samples or anomalies in the sensor readings, ensuring that the dataset is free from significant inconsistencies or errors. These steps were crucial to maintaining the high quality and reliability of the tactile data.

## Limitations

This study focuses on the collection of a tactile dataset specifically designed to explore textures on uneven surfaces, addressing a gap in current research. Unlike existing datasets, which predominantly focus on textures on flat or uniform surfaces, our dataset provides a unique resource for studying tactile perception in more complex scenarios. While the evaluation of different substrates beneath textures is indeed an intriguing and valuable avenue for future exploration, it falls beyond the scope of the present work.

By concentrating on uneven surfaces, our dataset contributes to advancing the understanding of tactile texture recognition in more challenging and realistic environments. The consideration of substrate effects and their influence on tactile data will be a subject for future research, building on the foundational work presented here.

## Ethics Statement

This research involving a tactile-enabled robotic manipulator utilized a BioIn-Tacto tactile sensing module. The study follows ethical guidelines outlined in the Guide for Authors. No human subjects were involved in this study. Therefore, informed consent and ethical committee approval were not required. Animal experiments were not conducted. Data collected from social media platforms was not used in this research. Therefore, participant consent and data redistribution policies were not applicable. The study focused on the technical aspects of the sensor module and robotic setup and did not involve any potential ethical concerns regarding human subjects, animal experiments, or social media data.

## CRediT Author Statement

**Maliheh Marzani:** Data collection, Validation, Data curation, Visualization, and Software. **Soheil Khatibi**: Validation, Data curation, Investigation, Writing- Original draft preparation and Software. **Ruslan Masinjila**: Writing- Reviewing and Editing, Validation, Data curation. **Vinicius Prado da Fonseca:** Supervision, Conceptualization, Writing- Reviewing and Editing, Validation, Data curation, and Funding acquisition. **Thiago Eustaquio Alves de Oliveira**, Supervision, Conceptualization, Writing- Reviewing and Editing, Validation, and Funding acquisition.

## Data Availability

Mendeley DataDynamic Tactile Data of Textures On Uneven Surfaces (Original data).
